# Azooxanthellate *Palythoa* (Cnidaria: Anthozoa) Genomes Reveal Toxin-related Gene Clusters and Loss of Neuronal Genes in Hexacorals

**DOI:** 10.1093/gbe/evae197

**Published:** 2024-09-06

**Authors:** Yuki Yoshioka, Hiroshi Yamashita, Taiga Uchida, Chuya Shinzato, Mayumi Kawamitsu, Chloé Julie Loïs Fourreau, Guillermo Mironenko Castelló, Britta Katharina Fiedler, Timotheus Maximilian van den Eeckhout, Stefano Borghi, James Davis Reimer, Eiichi Shoguchi

**Affiliations:** Marine Genomics Unit, Okinawa Institute of Science and Technology Graduate University, Onna, Okinawa 904-0495, Japan; Fisheries Technology Institute, Japan Fisheries Research and Education Agency, Ishigaki, Okinawa 907-0451, Japan; Atmosphere and Ocean Research Institute, The University of Tokyo, Kashiwanoha, Kashiwa 277-8564, Japan; Atmosphere and Ocean Research Institute, The University of Tokyo, Kashiwanoha, Kashiwa 277-8564, Japan; Sequencing Section, Okinawa Institute of Science and Technology Graduate University, Onna, Okinawa 904-0495, Japan; Molecular Invertebrate Systematics and Ecology (MISE) Lab, Graduate School of Engineering and Science, University of the Ryukyus, Nishihara, Okinawa, Japan; Molecular Invertebrate Systematics and Ecology (MISE) Lab, Graduate School of Engineering and Science, University of the Ryukyus, Nishihara, Okinawa, Japan; Molecular Invertebrate Systematics and Ecology (MISE) Lab, Graduate School of Engineering and Science, University of the Ryukyus, Nishihara, Okinawa, Japan; Molecular Invertebrate Systematics and Ecology (MISE) Lab, Graduate School of Engineering and Science, University of the Ryukyus, Nishihara, Okinawa, Japan; Institute for Biodiversity and Ecosystem Dynamics (IBED), University of Amsterdam, 1098 XH Amsterdam, The Netherlands; Molecular Invertebrate Systematics and Ecology (MISE) Lab, Graduate School of Engineering and Science, University of the Ryukyus, Nishihara, Okinawa, Japan; Biodiversity and Geosciences Program, Museum of Tropical Queensland, Queensland Museum Network, Townsville, QLD, Australia; College of Science and Engineering, James Cook University, Townsville, QLD, Australia; Molecular Invertebrate Systematics and Ecology (MISE) Lab, Graduate School of Engineering and Science, University of the Ryukyus, Nishihara, Okinawa, Japan; Tropical Biosphere Research Center, University of the Ryukyus, Nishihara, Okinawa, Japan; Marine Genomics Unit, Okinawa Institute of Science and Technology Graduate University, Onna, Okinawa 904-0495, Japan

**Keywords:** Zoantharia genome, gene loss, chitin degradation, prostaglandin biosynthesis, palytoxin, *MEGF11*

## Abstract

Zoantharia is an order among the Hexacorallia (Anthozoa: Cnidaria), and includes at least 300 species. Previously reported genomes from scleractinian corals and actiniarian sea anemones have illuminated part of the hexacorallian diversification. However, little is known about zoantharian genomes and the early evolution of hexacorals. To explore genome evolution in this group of hexacorals, here, we report de novo genome assemblies of the zoantharians *Palythoa mizigama* (Pmiz) and *Palythoa umbrosa* (Pumb), both of which are members of the family Sphenopidae, and uniquely live in comparatively dark coral reef caves without symbiotic Symbiodiniaceae dinoflagellates. Draft genomes generated from ultra-low input PacBio sequencing totaled 373 and 319 Mbp for Pmiz and Pumb, respectively. Protein-coding genes were predicted in each genome, totaling 30,394 in Pmiz and 24,800 in Pumb, with each set having ∼93% BUSCO completeness. Comparative genomic analyses identified 3,036 conserved gene families, which were found in all analyzed hexacoral genomes. Some of the genes related to toxins, chitin degradation, and prostaglandin biosynthesis were expanded in these two *Palythoa* genomes and many of which aligned tandemly. Extensive gene family loss was not detected in the *Palythoa* lineage and five of ten putatively lost gene families likely had neuronal function, suggesting biased gene loss in *Palythoa*. In conclusion, our comparative analyses demonstrate evolutionary conservation of gene families in the *Palythoa* lineage from the common ancestor of hexacorals. Restricted loss of gene families may imply that lost neuronal functions were effective for environmental adaptation in these two *Palythoa* species.

**Significance**

Anthozoan hexacorals are an important animal group in many marine environments, and include at least ∼3,500 extant species, including reef-building scleractinian corals. We generated two *Palythoa* genomes from the order Zoantharia within Hexacorallia, providing novel insights into early hexacorallian evolution by comparing with genomes of diversified scleractinian corals and actiniarian sea anemones. These first available gene-sets from zoantharians demonstrated genome conservation with restricted neuronal gene loss, and the suggested expansion of enzyme genes in *Palythoa* may be related to the production of unique chemicals and toxins such as palytoxin. Overall, our analyses imply that lineage-specific tandem duplication of enzyme genes may have occurred in the genome evolution of Zoantharia.

## Introduction

Hexacorallia, a cnidarian class of the subphylum Anthozoa, is a major and diversified group that includes the orders Scleractinia (stony corals), Corallimorpharia (mushroom anemones), Antipatharia (black corals), Actiniaria (true anemones), Zoantharia (colonial anemones), and Ceriantharia (tube anemones). Among these orders, Zoantharia is a sister group to a clade consisting of four orders: Actiniaria, Antipatharia, Corallimorpharia, and Scleractinia (McFadden et al. 2021). Scleractinia and Actiniaria have been comparatively well studied at the molecular level for a relatively long period of time (Shinzato and Yoshioka 2024). For example, Forêt et al. (2010) examined the genomes of scleractinians, discussing selective gene loss in Cnidaria, and proposed the conservation of ancestral genes in Anthozoa and lineage-specific gene families as the genomic basis for the diversification of stony corals (Yoshioka et al. 2022). Additionally, robust molecular studies on the origins of anthozoans have recently suggested that Hexacorallia had a common ancestor in the Cryogenian (711Ma), far older than had previously been estimated (McFadden et al. 2021). Among anemones, *Nematostella* and *Exaiptasia* have become model organisms for many genomic studies, and there are many data available for both species (Putnam et al. 2007; Baumgarten et al. 2015). Whole genomes of hexacorals have been reported from sea anemones and scleractinian corals (Putnam et al. 2007; Shinzato et al. 2011). However, genomic studies on zoantharians remain sparse, and this lack of information inhibits our ability to properly understand genomic evolution within the hexacorals.

Although genomes of zoantharians have been assembled with short reads (Santos et al. 2023), comparative analyses have been restricted to transposable elements (Fourreau et al. 2023), and thus far, gene models from genome assemblies remain unavailable. As well, whole transcriptome shotgun assemblies (TSA) of zoantharians have been analyzed, focusing on genes related to venoms and toxins (Huang et al. 2016; Liao et al. 2018, 2019). Although novel venom-related transcripts, novel functional toxins, and six groups of expressed peptide toxins were found (Liao et al. 2018), their genomic bases and regulatory mechanisms remain unclear.

Zoantharians can broadly be separated into two main suborders, although this classification remains controversial (McFadden et al. 2021; Fourreau et al. 2023). Members in the suborder Macrocnemina are found from shallow waters to the deep sea, and many species (but not all) are epibiotic, and as a group, they are known to be in symbioses with a wide variety of different marine phyla (Kise et al. 2023). On the other hand, the suborder Brachycnemina includes mostly shallow water tropical and subtropical species, with the large majority of species being zooxanthellate, in symbioses with Symbiodiniaceae (Davies et al. 2023). Among the Brachycnemina, the genera *Zoanthus* and *Palythoa* are the most speciose and well-known (Reimer et al. 2023), and are often common species in coral reef ecosystems (Reimer et al. 2023). *Palythoa* spp. have received research attention for their ecological role on coral reefs (Irei et al. 2015; Reimer et al. 2023), as well as their ability to produce palytoxin (PTX) (Deeds et al. 2011), one of the most potent toxins known from nature.

Recently, two closely related species of zoantharians with exceptional features, *Palythoa mizigama* and *Palythoa umbrosa*, were described from the Ryukyu Archipelago, Japan (Irei et al. 2015). These two species inhabit low-light environments such as coral reef caves and have no associations with photosymbiotic Symbiodiniaceae algae, unlike their congeners. As until now, no studies have reported on whether these species harbor PTX, and there have only been limited phylogenetic studies on them (Irei et al. 2015). As azooxanthellate and congeneric species, the loss of photoendosymbionts and their evolution to live in caves make them unique among *Palythoa*. Combined with the overall lack of zoantharian genomic information and these two species make good targets for investigating genomic evolution under such conditions.

**Fig. 1.**
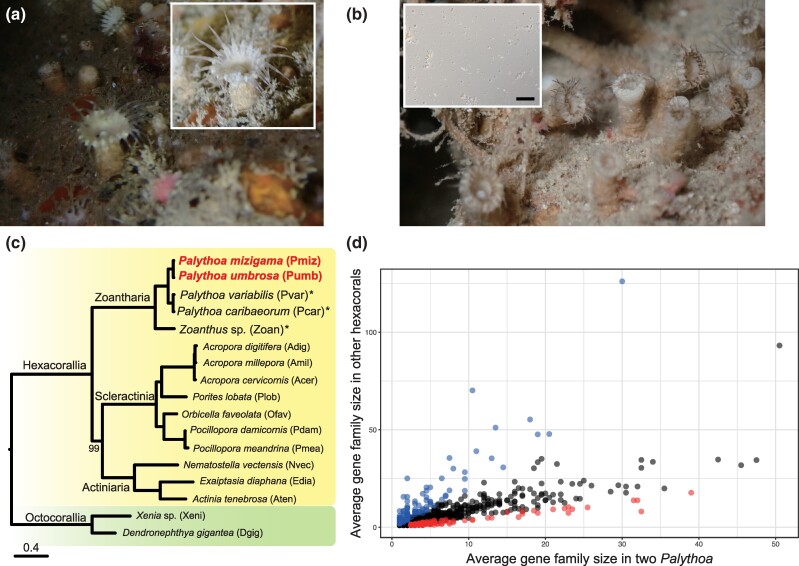
*P. mizigama* and *P. umbrosa* (Anthozoa: Hexacorallia: Zoantharia) in situ. a) A colony of *P. mizigama* in a marine cave. The specimens were used for genomic analyses in this study. The inset shows one illuminated polyp (∼5 mm in height). Image taken by C. J. L. Fourreau on October 9, 2023, at Mizugama. b) A colony of *P. umbrosa* used for the treatment of cell dissociations as the sample for genomic DNA extraction. The polyp is covered with grains of sand. Dissociated cells are indicated in the inset (Scale bar, 50 µm). Symbiodiniaceae cells were not found. c) Molecular phylogenetic tree of anthozoans constructed with 149 single copy orthologs. Asterisk indicates that genes are from transcriptome assemblies. Bootstrap value for each node was 100%, except one node with 99%. The bar indicates expected substitution per site in aligned regions. d) Gene families that were expanded or reduced in the Zoantharia lineage. Dots indicate 3,036 conserved gene families among 12 hexacoral genomes. Red and blue indicate their gene family sizes are two times larger or smaller than that of average in the other Hexacorallia.

Accordingly, here we report the whole-genome assembly of the two zoantharians, *P. mizigama* and *P. umbrosa* (Fig. 1a and b). By comparative genomic analyses, we provide insight into genome evolution of hexacorals by focusing on expanded gene families and putative gene loss. These genomes will also serve as the future basis for comparisons with zooxanthellate congeneric *Palythoa* spp.

## Results and Discussion

### Genome Assembly and Gene Models

We obtained 56 Gb of PacBio HiFi reads for *P. mizigama* and 53 Gb for *P. umbrosa* (supplementary table S1, Supplementary Material online). We successfully assembled complete mitochondrial genomes with a length of 21,122 bp and 21,145 bp, encoding 13 protein-coding genes for *P. mizigama* and *P. umbrosa*, respectively (supplementary fig. S1, Supplementary Material online).

After removing mitochondrial and contaminated HiFi reads, we performed nuclear genome assembly of the *Palythoa* species, resulting in draft genome assemblies of 373 and 319 Mb for *P. mizigama* and *P. umbrosa*, with mean depth of 102 to 107x, respectively (Table 1, supplementary fig. S2, Supplementary Material online). K-mer profiles suggested that estimated genome sizes for *P. mizigama* and *P. umbrosa* were ∼330 Mb and heterozygosity rate of ∼3.79% for *P. mizigama* and ∼3.39% for *P. umbrosa* (supplementary fig. S3, Supplementary Material online). The estimated genome sizes around 300 Mb were supported based on other k-mer profiles (Fourreau et al. 2023), suggesting that assembly sizes obtained in this study did not deviate from the expected sizes. When we compared assembly statistics with other zoantharian genomes, the numbers of contigs were significantly reduced and the indices of continuity (N50 and mean contig size) were significantly improved (supplementary table S2, Supplementary Material online). While single copy category in BUSCO completeness (Huang and Li 2023) in reported zoantharian genomes (Santos et al. 2023) were 17% to 49%, they were larger than 90% in our assemblies (supplementary table S2, Supplementary Material online). QV scores calculated with Inspector were over 50 (Table 1). These results indicate that the assemblies presented in this study are the first cases that achieved high continuity in zoantharian genomes.

**Table 1:** Statistics for genome assembly and gene prediction of *P. mizigama* and *P. umbrosa* with reported transcriptome shotgun assembly of zoantharians

Resource	Category	*Palythoa*				*Zoanthus*
		*P. mizigama*	*P. umbrosa*	*P. variabilis*	*P. caribaeorum*	*Zoanthus* sp.
Genome	Reference	This study	This study	Huang et al. 2016 (GCVI00000000.1)	Liao et al. 2018 (GESO00000000.1)	Liao et al. 2019 (GGTW00000000.1)
	No. of contigs	4,032	2,838	NA	NA	NA
	Assembly length (bp)	373,296,105	319,702,807	NA	NA	NA
	Min. length (bp)	1,026	1,034	NA	NA	NA
	Mean length (bp)	92,583	112,651	NA	NA	NA
	Max. length (bp)	1,310,536	1,179,232	NA	NA	NA
	N50 (bp)	248,916	237,614	NA	NA	NA
	Total gap length (bp)	0	0	NA	NA	NA
	GC contents (%)	37.20	36.95	NA	NA	NA
	QC score	50.1	50.8	NA	NA	NA
	Completeness	C: 93.92% (S: 92.56%, D: 1.36%) F: 2.73% I: 0.00% M: 3.35%	C: 92.87% (S: 92.24%, D: 0.63%) F: 2.73% I: 0.10% M: 4.30%	NA	NA	NA
Gene models/transcriptome assembly	No. of genes	30,394	24,800	NA	NA	NA
	Mean gene length (bp)	6,127	6,575	NA	NA	NA
	No. of transcripts	32,484	26,563	130,098	136,606	225,162
	Min. transcript length (bp)	102	102	200	200	200
	Mean transcript length (bp)	1,427	1,424	775	875	697
	Max. transcript length (bp)	60,264	48,444	10,958	26,101	24,409
	Completeness	C: 93.8% (S: 91.4%, D: 2.4%)^a^ F: 3.7% M: 2.5%	C: 91.8% (S: 90.9%, D: 0.9%)^a^ F: 4.5% M: 3.7%	C: 80.8% (S: 69.4%, D: 11.4%) F: 10.6% M: 8.6%	C: 84.9% (S: 73.2%, D: 11.7%) F: 8.8% M: 6.3%	C: 92.7% (S: 73.0%, D: 19.7%) F: 4.0% M: 3.3%

^a^The longest transcript variant per gene was used for the analysis.

We predicted 30,394 protein-coding genes for *P. mizigama* and 24,800 protein-coding genes for *P. umbrosa* (Table 1) based on protein-based gene prediction (see supplementary materials, Supplementary Material online). BUSCO completeness scores were 93.8% (of which 2.4% were duplicated) for *P. mizigama* and 91.8% (of which 0.9% were duplicated) for *P. umbrosa* (Table 1). This high BUSCO completeness score of gene models is comparable with those of gene models in the other cnidarians (supplementary table S3, Supplementary Material online), supporting acquisitions of high-quality gene models, and enabling more accurate comparative genomics to infer molecular bases of zoantharians.

### Toxin-Related Genes in *Palythoa* Genomes

**Fig. 2.**
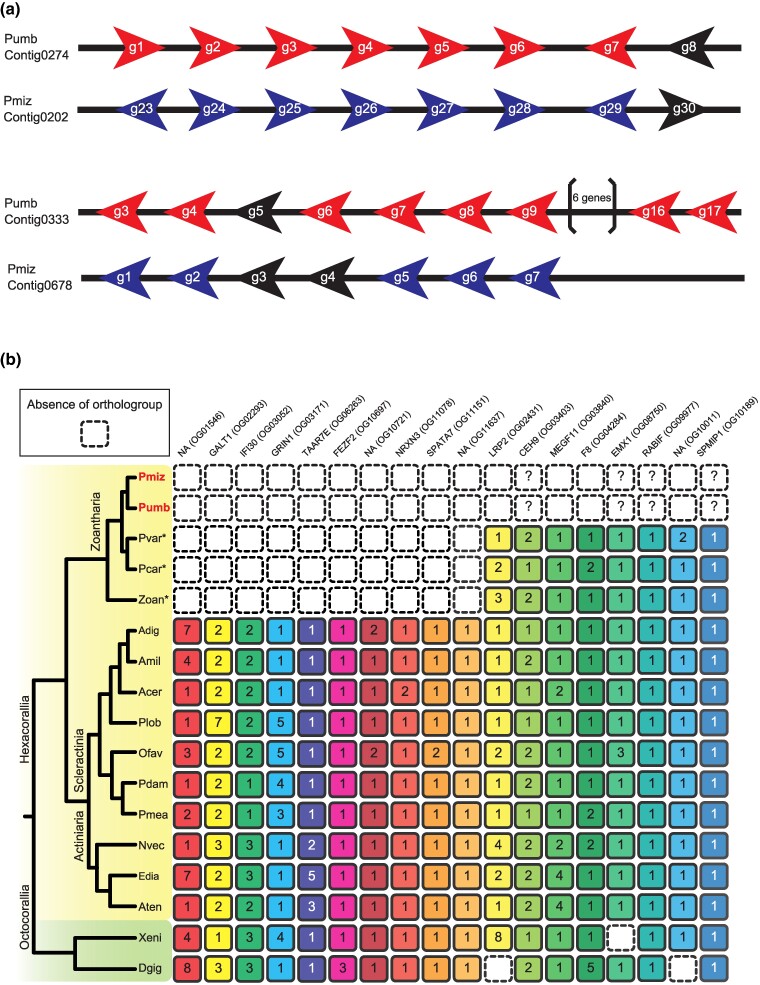
Tandem gene arrangement of toxin-related genes and putative gene loss in *Palythoa* lineage. a) Toxin-like genes belonging to orthogroup ID 000039 (Pumb contig0274 and Pmiz contig0202) and orthogroup ID 000067 (Pumb contig0333 and Pmiz contig0678). Contig ID is shown in the left and gene ID is shown in each arrowhead. Arrowhead indicates transcriptional direction. Red and blue indicate *P. umbrosa* and *P. mizigama* genes, respectively. Black indicates other genes (i.e. not related to toxin-like peptides). b) Possible gene name and orthogroup ID are shown in the upper row (NA indicates unknown gene families). Tree topology indicates phylogeny of Anthozoa (yellow shading: Hexacorallia; green shading: Octocorallia). Each box and number in the box indicates presence of the gene family and its copy number calculated with OrthoFinder. Boxes enclosed with dash indicate absence of the gene family. Question mark indicates detection by tBLASTn search against the genome, but no gene was predicted in the region. Detailed result of tBLASTn is shown in supplementary table S9, Supplementary Material online. Pmiz: *P. mizigama*, Pumb: *P. umbrosa*, Pvar: *P. variabilis*, Pcar: *P. caribaeorum*, Znat: *Zoanthus natalensis*, Adig: *Acropora digitifera*, Amil: *Acropora millepora*, Acer: *Acropora cervicornis*, Plob: *Porites lobata*, Pdam: *Pocillopora damicornis*, Pmea: *Pocillopora meandrina*, Nvec: *Nematostella vectensis*, Edia: *Exaiptasia diaphana*, Aten: *Actinia tenebrosa*, Xeni: *Xenia* sp., Dgig: *Dendronephthya gigantea*. Asterisk after species name indicates transcriptome assemblies. *GALT1*: *Beta-1,3-galactosyltransferase 1*, *IFI30*: *Gamma-interferon-inducible lysosomal thiol reductase*, *GRIN1*: *Glutamate receptor ionotropic*, *TAAR7E*: *Trace amine-associated receptor 7e*, *FEZF2*: *Fez family zinc finger protein 2*, *NRXN3*: *Neurexin-3*, *SPATA7*: *Spermatogenesis-associated protein 7*, *LRP2*: *Low-density lipoprotein receptor-related protein 2*, *CEH9*: *Homeobox protein ceh-9*, *MEGF11*: *Multiple epidermal growth factor-like domains protein 11*, *F8*: *Coagulation factor VIII*, *EMX1*: *Homeobox protein EMX1*, *RABIF*: *RAB interacting factor*, *SPMIP1*: *Sperm associated microtubule inner protein 1*.

Prior studies using transcriptome assemblies have focused on genes encoding toxin-like polypeptides (Huang et al. 2016; Liao et al. 2018, 2019). Putative toxin-related genes have been categorized into six main groups, with neurotoxin, hemostatic and hemorrhagic toxins, protease inhibitors, membrane-active peptides, mixed function enzymes, and peptides related to allergens and innate immunity components (Liao et al. 2018). We searched for their homologs in cnidarians based on orthogroup classification. Genes from each of these six groups were conserved in the other available cnidarian genomes (supplementary table S4, Supplementary Material online). Interestingly, some genes in these groups were tandemly arranged in both *P. mizigama* and *P. umbrosa* genomes (Fig. 2a; supplementary table S4, Supplementary Material online), suggesting that some of these tandem duplications occurred in the common ancestor of the genus *Palythoa*. Huang et al. (2016) suggested that putative toxins in *Palythoa* species are highly likely to be employed as an antipredatory armamentarium. Several species of fishes and turtles have been confirmed as predators of zoantharians (Stampar et al. 2007; Francini-Filho and Moura 2010). These duplications may enable rapid transcription of the genes when they face predators (Mathers et al. 2017).

The well-known toxin, PTX, has been detected in many different *Palythoa* specimens. It has been implied that polyketide synthases (PKSs) are related to the biosynthesis of PTX-like compounds (Verma et al. 2019). Our preliminary surveys of PKS genes found two PKS genes that encode multiple domain proteins (data not shown), suggesting no expansions of PKSs in these two *Palythoa* genomes. As the symbiosis-related function of a unique chemical that was biosynthesized with animal PKSs has been reported (Torres et al. 2020), zooxanthellate *Palythoa* spp. harboring symbiotic Symbiodiniaceae might have more than two PKS genes for the biosynthesis of unique chemicals (Deeds et al. 2011).

### Gene Family Expansion in *Palythoa* Lineage

In order to reveal the molecular basis underlying evolution of zoantharians, we inferred evolutionary relationships of genes among anthozoans (using genomes of two zoantharians [this study], seven scleractinians, three actiniarians, and two octocorallians as outgroup). Here, we included anthozoan genome assemblies available in RefSeq (supplementary table S3, Supplementary Material online). We used the orthogroups produced by OrthoFinder as putative gene families in this study. To gain a more comprehensive overview of gene families in *Palythoa* (zoantharians), we also included three transcriptome assemblies from *P. variabilis*, *P. caribaeorum*, and *Zoanthus* sp. in the analyses, resulting in a total of 66,871 gene families (supplementary data, Supplementary Material online). Of these, 3,036 gene families were conserved in the hexacorallian genomes, i.e. all hexacorallians used in this study possessed at least one gene per gene family, of which 149 were single copy, which were also conserved in the two octocorallian genomes as single copies (supplementary table S5, Supplementary Material online). Using the 149 single copy gene families, we performed molecular phylogenetic analyses. The tree topology was identical to the reported phylogeny of class Anthozoa (McFadden et al. 2021), and that five zoantharians formed a cluster, with two clear groups, *Palythoa* (*n* = 4) separate from the single *Zoanthus* (Fig. 1c).

Gene expansion (gene duplication) contributes to the evolution of organisms (Conant and Wolfe 2008). Using 3,036 conserved gene families, we identified gene families whose size (the number of paralogs) in the genus *Palythoa* were two-times different compared with that of the hexacorallian average. As transcriptome assemblies do not cover whole genes and there are difficulties in reducing redundancy (high duplicates BUSCO completeness, Table 1), we used *P. mizigama* and *P. umbrosa* as the representatives for the zoantharian lineage in the analyses. The sizes of 111 and 138 gene families in the two *Palythoa* genomes were two times larger or smaller than the average size in the other 10 hexacorallian genomes, respectively (Fig. 1d; supplementary tables S6 and S7, Supplementary Material online). Five functional categories, including immunity and cell adhesion, were identified by enrichment analysis as being significantly (FDR < 0.05) enriched in gene families with average sizes smaller than those in other hexacorallians (Table 2). In case of scleractinians, gene expansions for complex immune systems have previously been discussed to possibly be due to endosymbiosis with Symbiodiniaceae dinoflagellates (Shinzato et al. 2011). As our dataset included six scleractinians, increases of average gene family sizes of immune-related genes, including NOD-like receptors (OG00066), which have been shown to expand in a scleractinian coral lineage (Hamada et al. 2013), were observed (Fig. 1d, supplementary table S7, Supplementary Material online), confirming the reproducibility of the previous report. On the other hand, 10 functional categories, including peptide transport, chitin degradation, and prostaglandin biosynthesis, were identified by enrichment analysis as being significantly (FDR < 0.05) enriched in gene families with average sizes larger than those in other hexacorallians (Table 2). Diverse toxin-like peptides have been reported from zoantharians (Liao et al. 2019), and expansions of peptide transport may be related to the diversification of toxin-like peptides by tandem gene duplications in zoantharians (Fig. 2a). Chitin is the second-most abundant polysaccharide in nature (Tharanathan and Kittur 2003) and serves as a structural element of the exoskeleton of crustacean, and in cell walls in fungi and algae (Gooday 1990). Zoantharians are known to incorporate sand and/or detritus into their tissues to help strengthen their structure (Haywick and Mueller 1997), possibly increasing their encounters with fungal pathogens, as various components, including algae, accumulate more in sediment than in the water column (Littman et al. 2008; Amend et al. 2019). Chitinase can hydrolyze chitin into chitin oligosaccharides and/or monosaccharides and is widely distributed in marine organisms including scleractinian corals (Yoshioka et al. 2017), octocorals (Douglas et al. 2007), and *Palythoa caribaeorum* (Souza et al. 2008). The tentacle feeding response of *Palythoa* species, as well as of scleractinian corals, in the presence of zooplankton has been reported (Goreau et al. 1971), suggesting that they utilize chitinases to consume various zooplankton, such as copepods, with chitinous exoskeletons. As *P. mizigama* and *P. umbrosa* have no algal symbionts, utilization of duplicated chitinases might be beneficial to heterotrophically obtain their energy budget from plankton prey. In addition, possible functions of chitinase as protection against fungal pathogens in cnidarians have been hypothesized (Yoshioka et al. 2017; van de Water et al. 2018), suggesting that these chitin degradation-related genes (supplementary table S8, Supplementary Material online) may also act in defense systems in zoantharians, and that gene expansion of these genes may be related to the adaptive evolution of zoantharians in order to rapidly degrade invasive organisms with chitin via gene expression. It has been hypothesized that prostaglandins in octocorals could function as chemical defense against predators but this remains unclear (Di Costanzo et al. 2019). Some of these enzyme genes may be related to the production of unique chemicals and toxins in addition to prostaglandin biosynthesis.

**Table 2:** Differential biological process between zoantharians and other hexacorals predicted by gene family enrichment analysis

Category^a^	Biological process^b^	Fold enrichment	FDR
Larger	Amino-acid transport (KW-0029)	7.2	1.4709E-03
	Cell adhesion (KW-0130)	3.5	1.4709E-03
	Neurotransmitter biosynthesis (KW-0530)	41.5	1.4709E-03
	Prostaglandin metabolism (KW-0644)	18.5	1.8338E-03
	Polysaccharide degradation (KW-0624)	16.2	2.5023E-03
	Peptide transport (KW-0571)	29.6	2.5023E-03
	Chitin degradation (KW-0146)	18.9	9.5570E-03
	Prostaglandin biosynthesis (KW-0643)	17.3	1.0994E-02
	Symport (KW-0769)	4.2	2.0495E-02
	Transport (KW-0813)	1.5	2.1661E-02
Smaller	DNA integration (KW-0229)	40.6	3.5420E-13
	Innate immunity (KW-0399)	4.7	6.5366E-05
	Immunity (KW-0391)	4.0	6.5366E-05
	DNA recombination (KW-0233)	6.4	9.0097E-05
	Cell adhesion (KW-0130)	3.2	3.8349E-03

^a^Detailed gene annotation for “larger” and “smaller” are shown in supplementary tables S6 and S7, Supplementary Material online, respectively.

^b^ID for Uni-Prot keyword is shown in parentheses.

### Putative Gene Losses in *P. mizigama* and *P. umbrosa*

In addition to gene expansion, gene loss may also be related to adaptive phenotypic diversity and thus contributes to animal evolution (Albalat and Cañestro 2016). By comparing with representative hexacorallian genomes, we examined putative gene losses in the *Palythoa* lineage (Fig. 2b). Only 10 gene families were not detected in both genomes of *P. mizigama* and *P. umbrosa*, nor in available zoantharian transcriptome sets (*P. variabilis*, *P. caribaeorum*, and *Zoanthus* sp.; Fig. 2, supplementary table S9, Supplementary Material online), suggesting restrictive gene losses from the ancestral gene repertoire in the early zoantharian lineage. This result also suggests that genes involved in the biosynthesis of essential amino acids are conserved in the genomes of *P. mizigama* and *P. umbrosa* (Shinzato et al. 2011).

Interestingly, five out of the ten putative gene families that were putatively lost in zoantharian lineages; namely Glutamate receptor ionotropic (*GRIN1*), Trace amine-associated receptor 7e (*TAAR7E*), Fez family zinc finger protein 2 (*FEZF2*), Neurexin-3 (*NRXN3*), and Spermatogenesis-associated protein 7 (*SPATA7*), were neuronal genes (Kew and Kemp 2005; Shimizu et al. 2010; Liberles 2015; Dharmat et al. 2018; Zhang et al. 2023), indicating a biased pattern of gene loss (Albalat and Cañestro 2016). As species of *Palythoa* are known to possess PTX as potential neurotoxins (Fernández-Sánchez et al. 2021), the relationship between neuronal gene losses and gain of functions to retain toxins needs to be explored in the future.

Our detailed genomic analyses suggest the genomes of two *Palythoa* from dark coral reef environments have lost eight common gene families (Fig. 2). These events include the loss of *MEGF11*, of which orthologs play a critical role in the formation of retinal interneuron in humans (Kay et al. 2012).

The loss of genes involved in light sensing and neuronal function is a possible case of environmental variability in these *Palythoa* species that lack algal Symbiodiniaceae symbionts. Our results indicate that light recognition system appears to have retrogressed at least in *P. mizigama* and *P. umbrosa*. It should be noted that as some genes were also not detected in the three zoantharian transcriptome assemblies and as these three species are zooxanthellate, genomic information from other zooxanthellate zoantharians are needed for further discussion.

## Conclusions

In this study, we successfully obtained high quality gene models for two dark environment-adapted zoantharians, *P. mizigama* and *P. umbrosa*. Comparative genomic analyses among cnidarian anthozoans revealed putative gene losses, including a photoreceptor-related gene in both *P. mizigama* and *P. umbrosa*. Our genome assemblies identified expansions of enzyme genes related to prostaglandins and possibly toxins, although unique chemicals such as PTX still have not been identified in these two *Palythoa* species. For understanding zoantharians ecology and evolutionary success, further genomic information from sister-group species living in light environments will be needed and may illuminate new insight into genome evolution of hexacorals.

## Materials and Methods

The method details are shown in Supplementary material online at “Genome Biology and Evolution” online (Supplementary material online).

## Supplementary Material

evae197_Supplementary_Data

## Data Availability

Raw genomic DNA sequence data of *Palythoa mizigama* and *Palythoa umbrosa* have been submitted at DDBJ Sequence Read Archive (DRA) under the accession DRR546399-DRR546402 (BioProjectID: PRJDB18008), respectively. The genome assembly has been deposited at DNA Data Bank of Japan (DDBJ), the European Molecular Biology Laboratory (EMBL), and GenBank under the project Accession BAACCD010000001-BAACCD010004032 (*Palythoa mizigama* genome assembly) and BAACCE010000001-BAACCE010002838 (*Palythoa umbrosa* genome assembly). Gene models and contigs are also available from the Dryad: https://doi.org/10.5061/dryad.j0zpc86p9.
