# Supplementary material for: Azooxanthellate Palythoa (Cnidaria: Anthozoa) Genomes Reveal Toxin-related Gene Clusters and Loss of Neuronal Genes in Hexacorals
Source: Genome Biol Evol. 2024 Sep 6;16(9):evae197. doi: 10.1093/gbe/evae197 (PMC11413580; doi:10.1093/gbe/evae197)
Supplement: evae197_Supplementary_Data [file evae197_supplementary_data.zip › Yoshioka_et_al_Suppl_materials0717/Yoshioka_et_al_Suppl_materials0717/Yoshioka_et_al_Suppl_materials_rev0717.pdf]

Supplementary materials

Materials and methods

Sample collection

The two target species of the genus *Palythoa* (*P. mizigama* and *P. umbrosa*) were collected from different sites in southern Japan. *P. mizigama* was sampled at Mizugama, Okinawa Island, Okinawa, Japan on October 9, 2023 (Fig. 1A). The sampling location of *P. umbrosa* was at Nakano, Iriomote Island, Okinawa, Japan on September 16, 2023. Each *Palythoa* polyp was added into 50 mL tubes and they were chopped into small pieces with scissors. Fragmented tissues were put into 3.3x PBS and were kept for 30 min. By 5 min vortexing during this period, we dissociated the tissue into individual cells (Fig. 1B). Dissociated cells were centrifuged at 1,000 g for 5 min. After discarding the supernatant, they were quickly frozen in liquid nitrogen and were kept in a -80 °C freezer until processing.

DNA isolation and sequencing

High molecular weight DNAs from dissociated cells were extracted using innuPREP Plant DNA Kit I (Analytik Jena AG, Germany) following the manufacturer's protocol. Sequencing libraries from the genomic DNAs of each species were constructed according to HiFi Ultra-Low Input DNA protocol from Pacific Bioscience (Menlo Park, California, USA). The library construction included a whole-genome amplification step by PCR methods (Schneider et al. 2021) and some amplification bias may be caused. Sequencing was performed on the PacBio Sequel II systems (Pacific Bioscience, Menlo Park, CA, USA) using two SMRT (Single Molecule Real-Time) cells for each species.

Genome assembly and gene prediction

The mitochondrial genomes were first assembled from the HiFi reads with MitoHiFi pipeline v3.2.1 (Uliano-Silva et al. 2023). HiFi reads that mapped to the mitochondrial genomes were removed from further steps. HiFi reads possibly originating from archaea, bacteria, viral, plasmid, and UniVec Core were identified with KRAKEN2 v2.1.3 (Wood et al. 2019) and its standard database (January 2024 update) and were also removed from further steps. Prior to nuclear genome assembly, the k-mer profile (k=41 and 61) was performed with Meryl v1.4.1 (Rhie et al. 2020) and was used for genome size estimation with GenomeScope v2.0 (Ranallo-Benavidez et al. 2020). Contaminants- and mitochondrial-free HiFi reads were then assembled with Hifiasm v0.19.8-r603 (Cheng et al. 2021). Primary contigs assembled with Hifiasm were used for further analyses. Error-correction of the assembly was performed using Inspector v1.0.1 (Chen et al. 2021) with cleaned HiFi reads. Possible haplotypes were merged with HaploMerger2 v20180603

(Huang et al. 2017). This haplotype merging step was repeated for several times together with error collection using Inspector. Repetitive elements in the assembly were identified *de novo* with RepeatModeler v2.0.4 (Flynn et al. 2020) with option (-LTRStruct). Repeats identified *de novo* and known repeats in RepeatMasker database were concatenated and were soft-masked using RepeatMasker v4.1.5 (Smit et al. 2015). Simple tandem repeat detection was further performed by tantan v49 (Frith 2011). BUSCO completeness of the genome assembly was assessed with compleasm v0.2.5 (Huang and Li 2023) and "metazoa_odb10" database (n=954). To evaluate sequencing depth, cleaned HiFi reads were mapped to contigs with Minimap2 v2.26-r1175 and mean depth for each contig was calculated with BamDeal v0.27 (https://github.com/BGI-shenzhen/BamDeal?tab=readme-ov-file). GC content for each contig was calculated with SeqKit v2.8.0 (Shen et al. 2016). These were visualized using the "ggplot2" package (Ginestet 2011) in R v4.3.2 (R Foundation for Statistical Computing 2018).

Gene prediction was performed with BRAKER pipeline v3.0.8 (Hoff et al. 2019; Gabriel et al. 2021; Bruna et al. 2023). Softmasked genome and proteomes retrieved from OrthoDB v11 (metazoa) and proteomes from selected cnidarians registered in RefSeq ranging five orders (Supplementary table S2) were used as input in the pipeline. Gene with length larger than 100 bp were retained. Each gene was named 'g' plus a number from one end of each contig, e.g., c0001.g001 is the first gene of contig c0001. Gene models were assessed using BUSCO v5.7.0 (Manni et al. 2021) with "metazoa_odb10" database (2024-01-08, n=954). To ensure that no possible contamination was included, gene models were also assessed by OMArk v0.3.0 (Nevers et al. 2024) with OMAmer "Metazoa v2.0.0" database (n=3,244). Functional annotation of gene models were performed with BLASTP (E-value cutoff: 1e-3) (Camacho et al. 2009) against the Swiss-Prot database (7 June 2023). In addition, domains in protein sequences were searched with InterProScan v5.69-97.0 (Jones et al. 2014).

Orthogroup classification and molecular phylogenetic analyses

The longest transcript variants from each gene were selected from each organism and were translated into amino acids with TransDecoder v5.7.1 (https://github.com/TransDecoder/) as in (Yoshioka et al. 2022). Orthogroups were classified using OrthoFinder v2.5.5 (Emms and Kelly 2019) with the default option. Possible gene names for each gene family were assigned based on functional gene annotation of *P. umbrosa* or *Acropora millepora*. Protein sequences were aligned using MAFFT v7.520 (Katoh et al. 2002) with the option (--auto) and conserved region was retained using ClipKIT v2.2.4 (Steenwyk et al. 2020) with the option (-m kpi). Maximum likelihood analyses were performed using IQ-TREE v2.2.2.6 (Minh et al. 2020) with the option (-m MFP; -bb 1000). In the maximum likelihood analysis, ModelFinder (as packaged in IQ-TREE) (Kalyaanamoorthy et al. 2017) was used to identify the best-fit substitution model for each

gene region.

Enrichment analysis for gene families were performed on the web platform DAVID webserver (Sherman et al. 2022) with default settings. UniProt IDs from all genes of *P. umbrosa* were used as the background data set in the analysis, and UniProt IDs assigned to gene families whose gene family size were two-times different compared with that of average in hexacorallians were analyzed. UniProt keywords (biological process) representing False discovery rate (FDR) < 0.05 were considered significantly enriched terms.

To validate gene loss in *Palythoa* genomes, we performed tBLASTn search using the putative gene families against *P. umbrosa* and *P. mizigama* genomes.

References

Bruna T, Lomsadze A, Borodovsky M. 2023. GeneMark-ETP: Automatic Gene Finding in Eukaryotic Genomes in Consistency with Extrinsic Data. bioRxiv:2023.2001. 2013.524024.

Camacho C, Coulouris G, Avagyan V, Ma N, Papadopoulos J, Bealer K, Madden TL. 2009. BLAST+: architecture and applications. BMC bioinformatics 10:1-9.

Chen Y, Zhang Y, Wang AY, Gao M, Chong Z. 2021. Accurate long-read de novo assembly evaluation with Inspector. Genome biology 22:1-21.

Cheng H, Concepcion GT, Feng X, Zhang H, Li H. 2021. Haplotype-resolved de novo assembly using phased assembly graphs with hifiasm. Nature methods 18:170-175.

Emms DM, Kelly S. 2019. OrthoFinder: phylogenetic orthology inference for comparative genomics. Genome biology 20:1-14.

Flynn JM, Hubley R, Goubert C, Rosen J, Clark AG, Feschotte C, Smit AF. 2020. RepeatModeler2 for automated genomic discovery of transposable element families. Proceedings of the National Academy of Sciences 117:9451-9457.

Frith MC. 2011. A new repeat-masking method enables specific detection of homologous sequences. Nucleic acids research 39:e23-e23.

Gabriel L, Hoff KJ, Brůna T, Borodovsky M, Stanke M. 2021. TSEBRA: transcript selector for BRAKER. BMC bioinformatics 22:1-12.

Ginestet C. 2011. ggplot2: elegant graphics for data analysis. In: Oxford University Press.

Greiner S, Lehwark P, Bock R. 2019. OrganellarGenomeDRAW (OGDRAW) version 1.3.1: expanded toolkit for the graphical visualization of organellar genomes. Nucleic Acids Res. 47:W59–W64.

Hoff KJ, Lomsadze A, Borodovsky M, Stanke M. 2019. Whole-genome annotation with BRAKER. Gene prediction: methods and protocols:65-95.

Huang N, Li H. 2023. compleasm: a faster and more accurate reimplementation of BUSCO. Bioinformatics 39:btad595.

Huang S, Kang M, Xu A. 2017. HaploMerger2: rebuilding both haploid sub-assemblies from high-heterozygosity diploid genome assembly. Bioinformatics 33:2577-2579.

Jones P, Binns D, Chang H-Y, Fraser M, Li W, McAnulla C, McWilliam H, Maslen J, Mitchell A, Nuka G. 2014. InterProScan 5: genome-scale protein function classification. Bioinformatics 30:1236-1240.

Kalyaanamoorthy S, Minh BQ, Wong TK, Von Haeseler A, Jermiin LS. 2017. ModelFinder: fast model selection for accurate phylogenetic estimates. Nature methods 14:587-589.

Katoh K, Misawa K, Kuma Ki, Miyata T. 2002. MAFFT: a novel method for rapid multiple sequence alignment based on fast Fourier transform. Nucleic acids research 30:3059-3066.

Manni M, Berkeley MR, Seppey M, Simão FA, Zdobnov EM. 2021. BUSCO update: novel and streamlined workflows along with broader and deeper phylogenetic coverage for scoring of eukaryotic, prokaryotic, and viral genomes. Molecular biology and evolution 38:4647-4654.

Minh BQ, Schmidt HA, Chernomor O, Schrempf D, Woodhams MD, Von Haeseler A, Lanfear R. 2020. IQ-TREE 2: new models and efficient methods for phylogenetic inference in the genomic era. Molecular biology and evolution 37:1530-1534.

Nevers Y et al. 2024. Quality assessment of gene repertoire annotations with OMArk. Nat. Biotechnol. doi: 10.1038/s41587-024-02147-w.

R Foundation for Statistical Computing R. 2018. R: a language and environment for statistical computing. RA Lang Environ Stat Comput.

Ranallo-Benavidez TR, Jaron KS, Schatz MC. 2020. GenomeScope 2.0 and Smudgeplot for reference-free profiling of polyploid genomes. Nature communications 11:1432.

Rhie A, Walenz BP, Koren S, Phillippy AM. 2020. Merqury: reference-free quality, completeness, and phasing assessment for genome assemblies. Genome biology 21:1-27.

Schneider C et al. 2021. Two high-quality de novo genomes from single ethanol-preserved specimens of tiny metazoans (Collembola). Gigascience. 10. doi: 10.1093/gigascience/giab035.

Shen W, Le S, Li Y, Hu F. 2016. SeqKit: a cross-platform and ultrafast toolkit for FASTA/Q file manipulation. Plos One 11:e0163962.

Sherman BT, Hao M, Qiu J, Jiao X, Baseler MW, Lane HC, Imamichi T, Chang W. 2022. DAVID: a web server for functional enrichment analysis and functional annotation of gene lists (2021 update). Nucleic acids research 50:W216-W221.

Smit A, Hubley R, Green P. 2015. RepeatMasker Open-4.0. 2013–2015. https://www.repeatmasker.org/RepeatMasker/

Steenwyk JL, Buida III TJ, Li Y, Shen X-X, Rokas A. 2020. ClipKIT: a multiple sequence alignment trimming software for accurate phylogenomic inference. PLoS biology 18:e3001007.

Uliano-Silva M, Ferreira JGR, Krasheninnikova K, Formenti G, Abueg L, Torrance J, Myers EW, Durbin R, Blaxter M. 2023. MitoHiFi: a python pipeline for mitochondrial genome assembly from PacBio high fidelity reads. BMC bioinformatics 24:288.

Wood DE, Lu J, Langmead B. 2019. Improved metagenomic analysis with Kraken 2. Genome biology 20:1-13.

Yoshioka Y, Suzuki G, Zayasu Y, Yamashita H, Shinzato C. 2022. Comparative genomics highlight the importance of lineage-specific gene families in evolutionary divergence of the coral genus, Montipora. BMC Ecol Evol 22:71.

Supplementary figures:

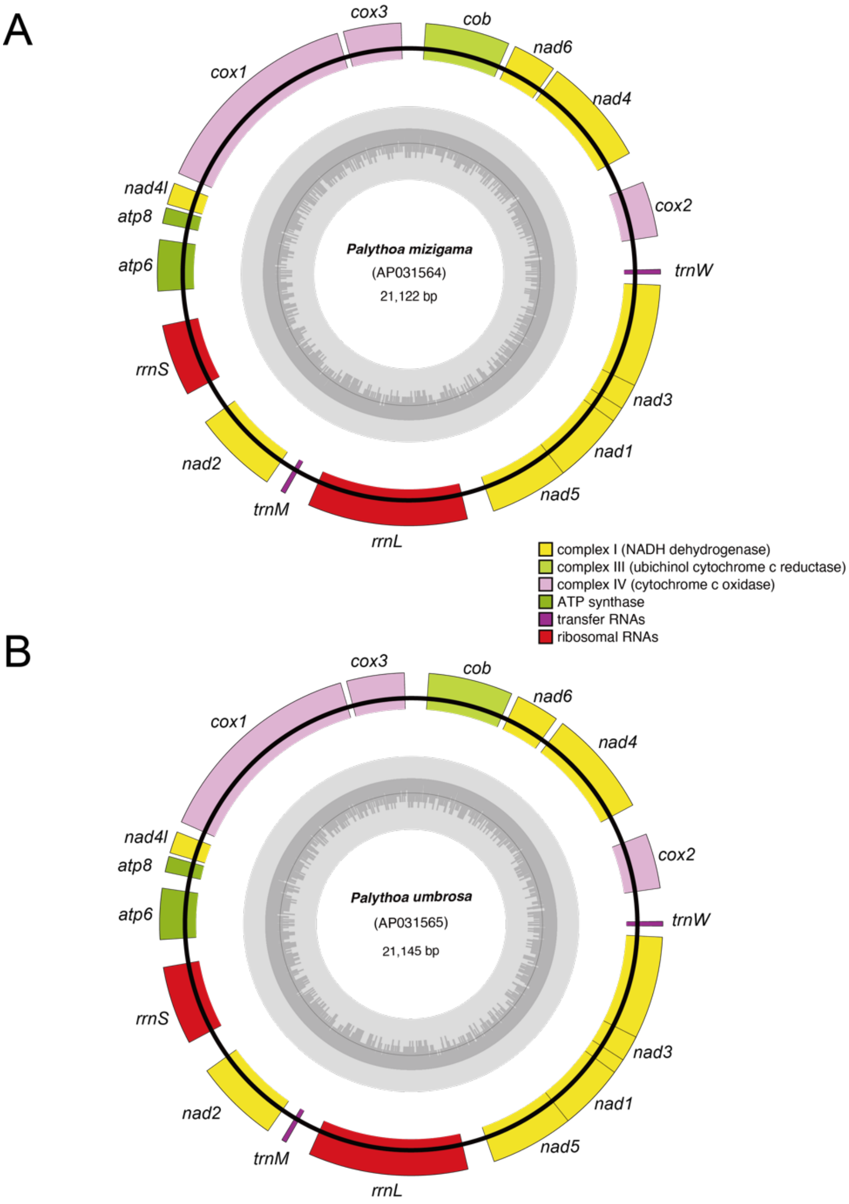

Supplementary fig. S1. Complete mitochondrial genomes of *P. mizigama* (A) and *P. umbrosa* (B). Figures visualized by OrganellarGenomeDRAW (OGDREW) (Greiner et al. 2019). Accession number and assembly length are shown on inside of the circle. Inner circles (grey) indicate GC contents. NADH dehydrogenase (yellow), ubiquinol cytochrome c reductase (light green), cytochrome c oxidase (pink), ATP synthase (green), tRNAs (purple), and rRNAs (red).

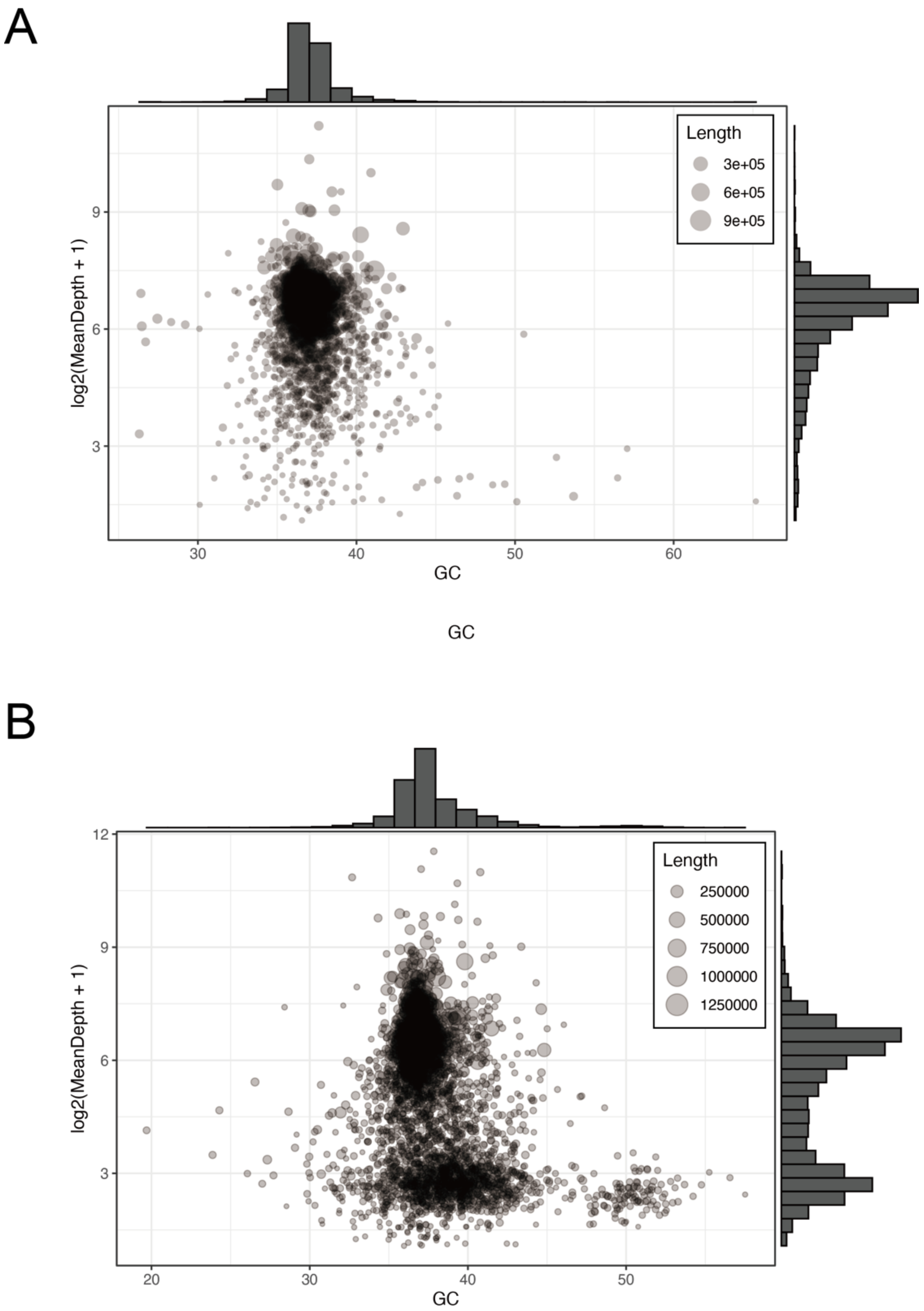

Supplementary fig. S2. Sequencing depth and GC contents in each contig.

P. umbrosa (A) and *P. mizigama* (B). Each dot indicates a single contig and their size corresponds to their contig length.

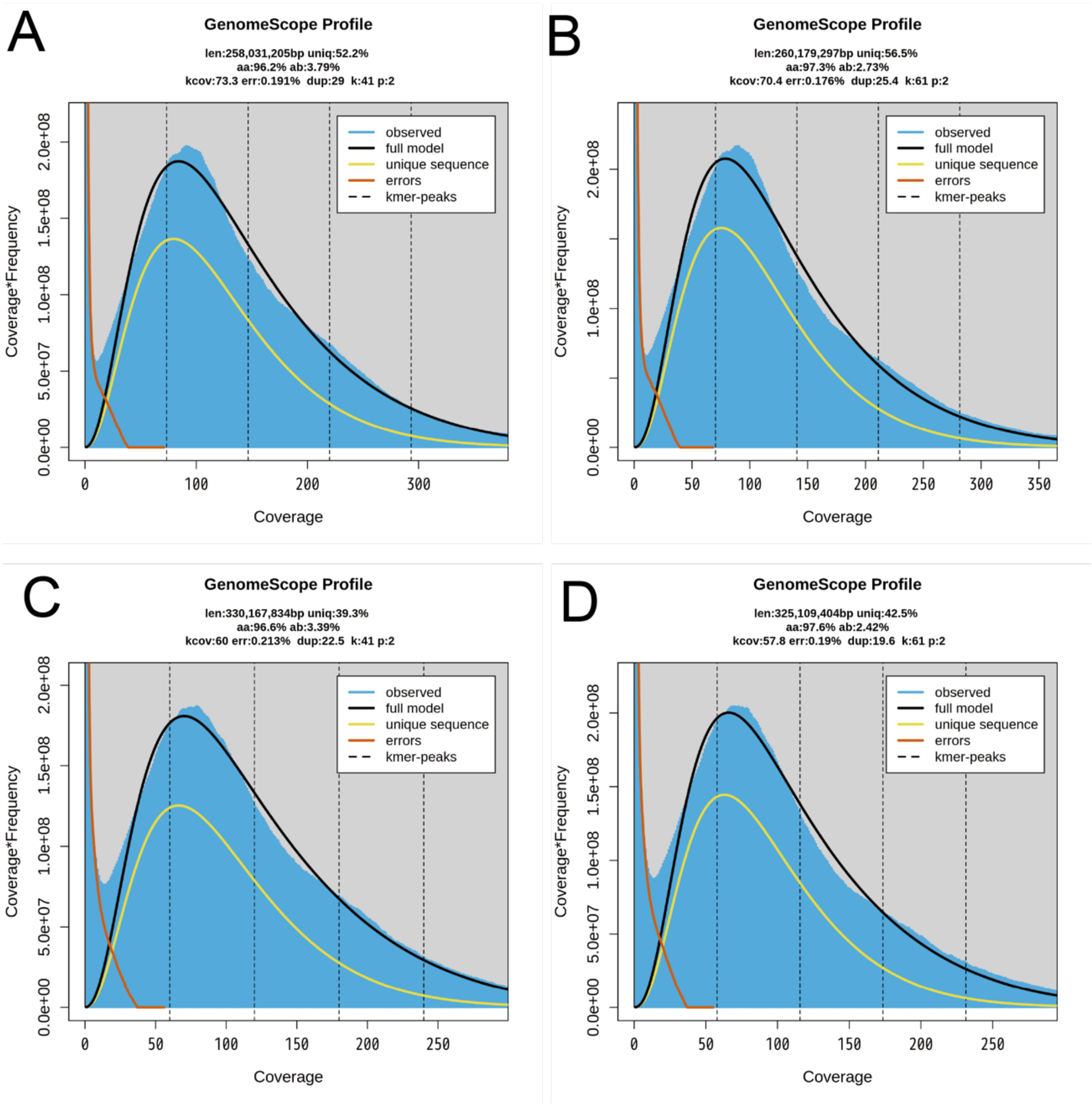

Supplementary fig. S3. Estimation of genome sizes.

Results of gene size estimation based on k-mer profiles for *P. umbrosa* with k-mer size 41 (A) and 61 (B) and for *P. mizigama* with k-mer size 41 (C) and 61 (D).

Supplementary tables:

Supplementary table S1. Summary of PacBio HiFi sequencing (long reads) and accession numbers.

Supplementary table S2. Statistics of genome assemblies among Zoantharia.

Supplementary table S3. List of proteomes used for gene prediction in this study.

Supplementary table S4. Identification of toxin- and venom-related genes based on previous report.

Supplementary table S5. Conserved gene families in Hexacorallia.

Supplementary table S6. Expanded gene families in both *P.mizigama* and *P. umbrosa* genomes.

Supplementary table S7. Smaller gene families in both *P.mizigama* and *P. umbrosa* genomes.

Supplementary table S8. Protein domains and sequence alignments of chitinases (OG0000581) in *P. umbrosa*.

Supplementary table S9. Genes possibly lost in *P. mizigama* and *P. umbrosa* genomes.
